# Innovation Support Reduces Quiet Quitting and Improves Innovative Behavior and Innovation Outputs among Nurses in Greece

**DOI:** 10.3390/nursrep14040193

**Published:** 2024-09-25

**Authors:** Ioannis Moisoglou, Aglaia Katsiroumpa, Ioanna Prasini, Parisis Gallos, Maria Kalogeropoulou, Petros Galanis

**Affiliations:** 1 Faculty of Nursing, University of Thessaly, 41500 Larisa, Greece; iomoysoglou@uth.gr; 2 Clinical Epidemiology Laboratory, Faculty of Nursing, National and Kapodistrian University of Athens, 11527 Athens, Greece; aglaiakat@nurs.uoa.gr (A.K.); parisgallos@nurs.uoa.gr (P.G.); mariakalo@nurs.uoa.gr (M.K.); 3 Palliative Care Unit Galilee, 19004 Spata, Greece; iprasini@galilee.gr

**Keywords:** innovation, behavior, nurses, quiet quitting scale, support, innovative behavior inventory, innovation outputs

## Abstract

Background: Innovation is a crucial issue in healthcare services since it can affect job-related variables such as productivity, satisfaction, and burnout. The aim of our study was to examine the impact of innovation support on quiet quitting, innovative behavior, and innovation outputs among nurses. Methods: We conducted a cross-sectional study in Greece during April 2024. We employed a convenience sample of nurses. We followed the reporting of observational studies in epidemiology (STROBE). We used the following instruments: (a) the innovation support inventory (ISI) to measure innovation support; (b) the quiet quitting scale (QQS) to measure quiet quitting; (c) the innovative behavior inventory (IBI) to measure innovative behavior; and (d) the innovation outputs (IO) scale to measure innovation outputs. Our study questionnaire was anonymous, and nurses gave their informed consent to participate. The Ethics Committee of the Faculty of Nursing, National and Kapodistrian University of Athens, approved our study protocol, while we followed the guidelines of the Declaration of Helsinki. Results: Our study population included 328 nurses with a mean age of 42.3 years (standard deviation: 9.7). Among them, 89.9% were females. Our multivariable analysis identified a negative relationship between innovation support and quiet quitting. Moreover, we found that managerial support and cultural support improved several aspects of innovative behavior, such as idea generation, idea search, idea communication, implementation starting activities, involving others, and overcoming obstacles. Additionally, managerial support improved innovation outputs. Conclusions: Our findings suggested the positive impact of innovation support on quiet quitting, innovative behavior, and innovation outputs among nurses. Organizations and nurses’ managers should establish an innovative working environment to improve nurses’ passion, motives, and productivity.

## 1. Introduction

Nurses who provide patient care face particular challenges in modern healthcare settings. These challenges include the increasing number of elderly patients with comorbidities, the requirement to enhance patient safety, the ongoing evolution of new health technologies, the constant need to integrate new scientific knowledge into daily clinical practice, the scarcity of available resources, and, in particular, the understaffing of services. In this highly demanding working environment, the development of innovative behavior by nurses can contribute to effective, efficient, and quality patient care.

A generally accepted definition of innovation is “the intentional introduction and application within a role, group, or organization of ideas, processes, products, or procedures, new to the relevant unit of adoption, designed to significantly benefit the individual, the group, the organization, or wider society” [1]. As healthcare organizations are complex, there is plenty of space to implement innovative actions. Among the four key roles of nurses, the American Nurses Association (ANA) includes that of nurses as innovators [2]. Nurses’ innovative behaviors include idea generation, idea search, idea communication, implementation initiation activities, overcoming obstacles, innovation outputs, and engaging others [3]. Over time, nurses act as agents of innovation, positively influencing both the quality of healthcare and policy issues related to their profession. In particular, the implementation of innovative nursing protocols has contributed to the reduction of nosocomial infections, medication errors, and the better management of chronic diseases [4,5,6]. Nurses have shown a positive attitude and have adopted health information technology applications, such as the electronic health record or the use of mobile phone applications, in order to improve the quality of care provided to their patients [7,8,9]. In terms of health policies, innovative initiatives by nurses have contributed to the implementation of changes to improve nursing education [10].

For nurses to develop innovative behaviors, as well as to receive support and promotion of such work behaviors, the importance of their work environment has been recognized. Elements of the nurses’ work environment, such as the foundation of quality, good working relationships between nurses and physicians, support from the supervisor and the organization, and the organizational culture, enhance the manifestation of innovative behavior [11,12,13]. The most crucial aspect in the growth of nurses’ innovative behavior is the support they receive from their immediate supervisor, particularly in terms of leadership. An inclusive leadership style, where an individual has the ability to lead a diverse group of people while showing respect for each person’s unique characteristics without prejudice, was found to have a significantly positive effect on innovative behavior [14]. One leadership style that provides opportunities for nurses to express new ideas and take initiatives is that of transformational leadership. A study has shown that transformational leadership was the most influential and also a predictor of innovative work behavior in nurses, compared to other leadership styles [15]. The supervisor’s endorsement of innovation and the nurses’ innovative behavior both impact the quality of nursing care and the well-being of the nurses [16,17]. In addition, the supervisor’s demonstration of an ethical, humanistic, empathic, and mutually beneficial approach, as well as adopting a servant leadership strategy, encourages nurses to engage in innovative behavior [18].

An alarming trend that emerged during the COVID-19 pandemic is the behavior of quiet quitting. The concept of quiet quitting refers to a situation where an employee does not formally resign from his/her job but instead reduces level of performance. Employees only meet the basic criteria of the job without exerting extra effort, working longer hours, or arriving earlier, and without going above and beyond what is expected [19]. A comprehensive survey conducted in the United States by Gallup, a prominent job analysis and consulting agency, revealed that 50% of American corporate employees had chosen to quiet quit [20]. The primary factors contributing to employees quiet quitting work behavior include the employer’s insufficient dedication to the advancement of employees’ careers, management’s failure to acknowledge the worth of their subordinates, a growing disengagement of employees from their work, a lack of recognition for the significance of employees’ autonomy, and a decline in organizational trust among employees [21]. The development of a reliable and valid questionnaire made it possible to study this phenomenon in all sectors [22,23]. Initial research in the healthcare industry revealed the magnitude of the problem, indicating that nurses are more likely to engage in quiet quitting compared to other healthcare professionals, with a rate exceeding 60% [24]. Quiet quitting can be influenced by burnout and bullying, which are recognized as contributing factors. On the other hand, emotional intelligence and moral resilience decrease the chance of this behavior. Nurses who opt for quiet quitting are more inclined to express their turnover intention from their position [25]. The choice of quiet quitting by nurses is a work behavior that can be a barrier to innovation and efficiency in an organization, as these employees have no commitment to the organization, their thoughts are on leaving their job, and they do not show willingness to go beyond their job.

To the best of our knowledge, there are no studies that investigated the relationship between innovation support and quiet quitting, while the study of the relationship between innovation support and innovative behavior and innovation outputs among nurses is limited [13,26,27]. In particular, two studies in Turkey [13,26] and one study in Iran [27] revealed the positive impact of innovation support on innovative behavior and innovation outputs among nurses. All studies included nurses working in hospitals. Thus, our aim was to explore the impact of innovation support on quiet quitting, innovative behavior, and innovation outputs in a sample of nurses. In this context, our research hypotheses were the following:

**H1.**  *Is there an association between innovation support and quiet quitting?*

**H2.**  *Is there an association between innovation support and innovative behavior?*

**H3.**  *Is there an association between innovation support and innovation outputs?*

## 2. Materials and Methods

### 2.1. Study Design

We conducted an online cross-sectional study in Greece during April 2024. Our inclusion criteria were the following: (a) nurses who understand the Greek language and (b) nurses who have been working at least three years in a clinical setting. We excluded nurses that do not understand the Greek language and do not work in clinical settings. We followed the reporting of observational studies in epidemiology (STROBE) [28].

### 2.2. Sample and Data Collection

We obtained a convenience sample of nurses through social media. In particular, we created an online version of our study questionnaire with Google Forms. Then, we posted the questionnaire on nurses’ groups on Facebook and LinkedIn. We posted the questionnaire three times during a month to remind nurses to participate in our study. Our study questionnaire was anonymous, and nurses gave their informed consent to participate. In particular, we informed nurses about the aim and design of our study through the online questionnaire. After that, we asked nurses if they wanted to participate in our study. Nurses with a positive answer can then proceed to answer our questionnaire.

Minimum sample size was 262 nurses, assuming a low effect size (f^2^ = 0.05) between predictors (i.e., managerial support, organizational support, and cultural support) and dependent variables (i.e., quiet quitting, innovative behavior, and innovation outputs), power of analysis as 95%, alpha level as 5%, and number of independent variables as 8 (i.e., three predictors and five confounders) [29].

### 2.3. Instruments

We measured demographic and job characteristics of nurses as follows: gender (females or males), age (continuous variable), understaffed department (no or yes), shift work (no or yes), and work experience (continuous variable). Additionally, we used the following instruments:

Innovation support inventory (ISI) [30]: The ISI includes 12 items and three factors: (a) “managerial support” with five items (item example: “My manager motivates me to come to him/her with new ideas”), (b) “organizational support” with three items (item example: “The way of remuneration in our organization motivates employees to suggest new things and procedures”), and (c) “cultural support” with four items (item example: “Most people in Greece come up with new, original ideas at work”). Answers are on a 5-point Likert scale from 1 (totally disagree) to 5 (totally agree). The score for each factor ranges from 1 to 5. Higher values represent more innovation. We used the valid Greek version of the ISI [31]. In our study, Cronbach’s alpha for “managerial support”, “organizational support”, and “cultural support” was 0.825, 0.701, and 0.727, respectively.

Quiet quitting scale (QQS) [23]: The QQS includes nine items and three factors: (a) “detachment” with four items (item example: “I do the basic or minimum amount of work without going above and beyond”), (b) “lack of initiative” with three items (item example: “I do not express opinions and ideas about my work because I think that work conditions are not going to change”), and (c) “lack of motivation” with two items (item example: “I do not find motives in my job”). Answers are on a 5-point Likert scale from 1 (strongly disagree) to 5 (strongly agree). The score for each factor ranges from 1 to 5. Higher values represent higher levels of quiet quitting. We used the valid Greek version of the QQS [24]. Scale developers suggest a cut-off point of 2.06 to distinguish quiet quitters from non quiet quitters [22]. In our study, Cronbach’s alpha for “detachment”, “lack of initiative”, and “lack of motivation” was 0.788, 0.729 and 0.861, respectively.

Innovative behavior inventory (IBI) [30]: The IBI includes 20 items and six factors: (a) “idea generation” with three items (item example: “I try new ways of doing things at work”), (b) “idea search” with three items (item example: “I try to get new ideas from colleagues or business partners”), (c) “idea communication” with four items (item example: “When I have a new idea, I try to persuade my colleagues of it”), (d) “implementation starting activities” with three items (item example: “I develop suitable plans and schedules for the implementation of new ideas”), (e) “involving others” with three items (item example: “I try to involve key decision makers in the implementation of an idea), and (f) “overcoming obstacles” with four items (item example: “I do not give up even when others say it cannot be done”). Answers are on a 5-point Likert scale from 1 (fully disagree) to 5 (fully agree). Score on each factor ranges from 1 to 5. Higher values represent more innovation. We used the valid Greek version of the IBI [31]. In our study, Cronbach’s alpha for “idea generation”, “idea search”, “idea communication”, “implementation starting activities”, “involving others”, and “overcoming obstacles” was 0.723, 0.860, 0.873, 0.818, 0.790, and 0.885, respectively.

Innovation outputs (IO) scale [30]: The IO includes three items and one factor. Item examples are the following: “I was often successful at work in implementing my ideas and putting them in practice” and “Many things I came up with are used in our organization”. Answers are on a 5-point Likert scale from 1 (totally disagree) to 5 (totally agree). Score ranges from 1 to 5. Higher values represent better innovation outputs. We used the valid Greek version of the IO [31]. In our study, Cronbach’s alpha for the IO was 0.706.

### 2.4. Ethical Issues

The Ethics Committee of the Faculty of Nursing, National and Kapodistrian University of Athens (reference number: 498, 1 April 2024) approved our study protocol. Additionally, our study followed the guidelines of the Declaration of Helsinki [32].

### 2.5. Statistical Analysis

We use numbers and percentages to present categorical variables. Moreover, we use mean, standard deviation (SD), median, minimum value, and maximum value to present continuous variables. We applied the Kolmogorov–Smirnov test and Q-Q plots to assess the distribution of continuous variables. We found that all continuous variables followed normal distribution. Innovation support was the independent variable, while quiet quitting, innovative behavior, and innovation outputs were the dependent variables. Since our dependent variables were continuous variables that followed normal distribution, we performed linear regression analysis. First, we conducted univariate regression analysis to examine the impact of innovation support without taking into consideration confounders. Then, we considered our demographic and job characteristics as potential confounders in the relationship between innovation support and quiet quitting, innovative behavior, and innovation outputs. In that way, we finally constructed multivariable linear regression models by eliminating confounders. We present unadjusted and adjusted coefficients beta, 95% confidence intervals (CI), *p*-values, and R2. *p*-values less than 0.05 were considered statistically significant. Since age and work experience were highly correlated (Pearson’s correlation coefficient: 0.919; *p* < 0.001), we included only age in the multivariable models to avoid multicollinearity. We used the IBM SPSS 21.0 (IBM Corp.), released in 2012, and IBM SPSS Statistics for Windows, version 21.0 (IBM Corp., Armonk, NY, USA) for the analysis.

## 3. Results

### 3.1. Demographic and Job Characteristics

Our study population included 328 nurses. Among them, 89.9% (n = 295) were females and 10.1% (n = 33) were males. The mean age of our nurses was 42.3 years (SD: 9.7) with a range from 22 to 60 years. In our sample, 81.1% (n = 266) reported that they have been working in understaffed departments, and 70.1% (n = 230) have been working in shifts. The mean years of work experience was 17.7 (SD: 10.2), with a range from 3 to 36 years.

### 3.2. Study Scales

Detailed descriptive statistics for the study scales are shown in Table 1. Nurses reported higher levels of cultural support (mean: 2.94; SD: 0.66) and managerial support (mean: 2.61; SD: 0.79) than organizational support (mean: 1.98; SD: 0.76).

Lack of motivation (mean: 2.90; SD: 1.03) was higher than lack of initiative (mean: 2.39; SD: 0.85) and detachment (mean: 2.00; SD: 0.73). Among our nurses, 66.2% (n = 217) could be considered quiet quitters since they had a score on QQS above the cut-off point of 2.06.

Regarding innovative behavior, nurses reported higher values on “idea search” (mean: 3.98; SD: 0.69) and “involving others” (mean: 3.64; SD: 0.69), and then on “idea generation” (mean: 3.60; SD: 0.63), “idea communication” (mean: 3.60; SD: 0.73), “overcoming obstacles” (mean: 3.56; SD: 0.76), and “implementation starting activities” (mean: 3.26; SD: 0.79).

The mean value of “innovation outputs” was 3.46 (SD: 0.65) with a range from 1.3 to 5.

### 3.3. Quiet Quitting

Our multivariable linear regression models identified a negative relationship between innovation support and quiet quitting (Table 2). In particular, managerial support reduced detachment (adjusted coefficient beta = −0.173; 95% CI = −0.281 to −0.065; *p*-value = 0.002), lack of initiative (adjusted coefficient beta = −0.314; 95% CI = −0.435 to −0.194; *p*-value < 0.001) and lack of motivation (adjusted coefficient beta = −0.331; 95% CI = −0.469 to −0.192; *p*-value < 0.001).

Additionally, organizational support (adjusted coefficient beta = −0.187; 95% CI = −0.334 to −0.039; *p*-value = 0.014) and cultural support (adjusted coefficient beta = −0.288, 95% CI = −0.446 to −0.130; *p*-value < 0.001) reduced lack of motivation.

### 3.4. Innovative Behavior

Linear regression models with innovative behavior as the dependent variable are shown in Table 3. We found a positive relationship between innovation support and innovative behavior. In particular, managerial support improved several aspects of innovative behavior, such as idea generation (adjusted coefficient beta = 0.163; 95% CI = 0.072 to 0.253; and *p*-value < 0.001), idea search (adjusted coefficient beta = 0.119; 95% CI = 0.016 to 0.222; and *p*-value = 0.023), idea communication (adjusted coefficient beta = 0.207; 95% CI = 0.107 to 0.306; and *p*-value < 0.001), implementation starting activities (adjusted coefficient beta = 0.221; 95% CI = 0.112 to 0.330; and *p*-value < 0.001), involving others (adjusted coefficient beta = 0.154; 95% CI = 0.057 to 0.252; and *p*-value = 0.002), and overcoming obstacles (adjusted coefficient beta = 0.216; 95% CI = 0.107 to 0.324; and *p*-value < 0.001).

Moreover, we found that cultural support has a positive impact on idea generation (adjusted coefficient beta = 0.181; 95% CI = 0.078 to 0.284; and *p*-value = 0.001), idea communication (adjusted coefficient beta = 0.149; 95% CI = 0.036 to 0.262; and *p*-value = 0.010), and implementation starting activities (adjusted coefficient beta = 0.147; 95% CI = 0.023 to 0.272; and *p*-value = 0.020).

### 3.5. Innovation Outputs

Managerial support improved innovation outputs (adjusted coefficient beta = 0.230; 95% CI = 0.144 to 0.317; and *p*-value < 0.001), while organizational support and cultural support did not affect innovation outputs (Table 4).

## 4. Discussion

This study investigated the role of innovative support in the emergence of quiet quitting as a work behavior and the effect of this support on the development of innovative behavior and the occurrence of innovation outcomes. The study findings indicated that the majority of participants are quiet quitters (experience detachment, lack of initiative, and lack of motivation), exhibit innovative behavior, and providing support for innovation decreases the chances of quiet quitting, promotes innovative behavior, and improves innovative results. Research on quiet quitting in the international health sector is fairly sparse, but our findings align with previous studies. A study conducted with 1760 healthcare professionals revealed that the proportion of nurses choosing quiet quitting was 66.4%, which was the greatest compared to other medical staff and healthcare professionals [24]. In another study, almost 77% of nurses were shown to demonstrate quiet quitting [33]. Detachment, lack of initiative, and lack of motivation are the three factors that compose quiet quitting. These factors can hinder an employee’s ability to exhibit innovative behavior. The expression of personal initiative by nurses is associated with both idea generation and concept implementation [34]. By implementing the transformational leadership style, nurse supervisors have the ability to positively impact the psychological empowerment of nurses. This, in turn, has an influence on both intrinsic motivation and information sharing behavior, ultimately leading to an enhancement in innovative work behavior [35]. While all four attributes of transformational leadership (idealized influence, inspiration, intellectual stimulation, and individualized consideration) contribute positively to the growth of employees and the organization, it is intellectual stimulation in particular that fosters greater innovation and creativity among followers [36]. Also, there is a direct correlation between the growth in nurses’ work engagement and their level of innovative behavior [37].

The participants in the current study had elevated scores in terms of innovative behavior. Aside from leadership support, particularly from supervisors, numerous other aspects in the nurses’ work environment have a key role in fostering innovative behavior. Research findings indicate that higher levels of education and possession of certifications have a beneficial impact on an individual’s innovative behavior [38]. Hence, the hospital administration’s facilitation of educational and ongoing training opportunities for nurses might be considered as measures aimed at fostering innovation. Moreover, by enabling nurses to access information and fostering a culture of learning inside the organization, it not only establishes a structure for ongoing improvement and professional growth to enhance the quality of care but also encourages nurses to engage in innovative behavior [39,40]. The level of professional autonomy among nurses plays a significant role in fostering innovation within a health organization. Nurses who have a high degree of autonomy are able to generate innovative outcomes and contribute additional value through their innovative practices in delivering patient care and overall health services [41].

The present study also highlighted the impact of cultural support on reduced lack of motivation and the positive impact on idea generation, idea communication, and initiation of implementation activities. Most research focuses limitedly on the influence of managerial and organizational support on innovation. The significance and influence of national culture are somewhat undervalued, despite the fact that national culture serves as the framework within which organizations evolve. The findings of our study align with the existing body of international research, which has emphasized the impact of national culture on organizational culture [42,43]. Consumer innovativeness and innovation adoption behavior are frequently influenced by the cultural context of a country [44]. Efforts are already being made in the health sector to create a brand-new, comprehensive ecosystem for health innovation that influences how the public and commercial sectors collaborate in mutually beneficial partnerships to provide equal access as the main goal [45].

The health services industry is a dynamic setting marked by the growing occurrence of chronic illnesses, the need for ongoing enhancement of service safety, and the constant development of biomedical technology. The implementation of innovative approaches by nurses effectively tackles these issues and ensures the provision of optimal care [46,47,48]. The continuous backing from nursing leadership has the potential to augment the innovative endeavors of nurses [49]. Continual support for nurses is necessary due to their provision of services in a challenging work environment characterized by elevated levels of dissatisfaction, burnout, and quiet quitting [24,50]. An inadequate level of occupational wellbeing among nurses can hinder the emergence of creative conduct. Nurse leadership has the potential to amplify innovation, creativity, and well-being within the healthcare sector [51].

Our study had several limitations. First, we cannot establish a causal relationship between innovation support, quiet quitting, innovative behavior, and innovation outputs since we conducted a cross-sectional study. Second, we employed a convenience sample through social media to collect our data. Thus, our sample cannot be representative of the nurse’s population in Greece. Although we achieved the minimum required sample size, further studies with random and more representative samples should be conducted not only in Greece but worldwide. Third, we eliminated several confounders in the relationship between innovation support and quiet quitting, innovative behavior, and innovation outputs. However, several other variables can act as confounders on this relationship and should be investigated in the future to get more valid results. Finally, although we used valid instruments to measure innovation support, quiet quitting, innovative behavior, and innovation outputs, the self-reported nature of these can introduce information bias in our study.

## 5. Conclusions

Innovation is a crucial component for improving a healthcare organization. The nurses’ innovative behavior, as front-line healthcare providers, can enhance the quality of care and thus increase the organization’s efficiency. This study emphasized the innovative behavior of nurses and the pivotal impact of management on the cultivation of this behavior and its results. Furthermore, healthcare organizations must not only promote innovation but also tackle the issue of quiet quitting, which was identified as having a significant impact in this study and may hinder the development of innovative behavior among nurses.

## Figures and Tables

**Table 1:** Descriptive statistics for the study scales (N = 328).

Scale	Mean	Standard Deviation	Median	Minimum Value	Maximum Value
Innovation support inventory					
Managerial support	2.61	0.79	2.60	1	5
Organizational support	1.98	0.76	2.00	1	5
Cultural support	2.94	0.66	3.00	1	5
Quiet quitting scale					
Detachment	2.00	0.73	2.00	1	4.5
Lack of initiative	2.39	0.85	2.33	1	4.7
Lack of motivation	2.90	1.03	3.00	1	5
Innovative behavior inventory					
Idea generation	3.60	0.63	3.67	1.3	5
Idea search	3.98	0.69	4.00	1	5
Idea communication	3.60	0.73	3.75	1	5
Implementation starting activities	3.26	0.79	3.33	1	5
Involving others	3.64	0.69	3.67	1	5
Overcoming obstacles	3.56	0.76	3.50	1.3	5
Innovation outputs	3.46	0.65	3.67	1.3	5

**Table 2:** Linear regression models with quiet quitting as the dependent variable (N = 328).

Dependent Variable *Independent Variables*	Univariate Model			Multivariable Model ^a^		
	Unadjusted Coefficient Beta	95% CI for Beta	*p*-Value	Adjusted Coefficient Beta	95% CI for Beta	*p*-Value
Detachment ^b^						
*Managerial support*	−0.136	−0.235 to −0.036	0.008	−0.173	−0.281 to −0.065	0.002
*Organizational support*	0.047	−0.057 to 0.152	0.373	0.012	−0.132 to 0.189	0.563
*Cultural support*	−0.032	−0.153 to 0.088	0.599	−0.036	−0.159 to 0.087	0.565
Lack of initiative ^c^						
*Managerial support*	−0.335	−0.446 to −0.223	<0.001	−0.314	−0.435 to −0.194	<0.001
*Organizational support*	−0.190	−0.311 to −0.069	0.002	−0.038	−0.167 to 0.090	0.560
*Cultural support*	−0.116	−0.256 to 0.025	0.105	−0.037	−0.175 to 0.100	0.592
Lack of motivation ^d^						
*Managerial support*	−0.460	−0.592 to −0.327	<0.001	−0.331	−0.469 to −0.192	<0.001
*Organizational support*	−0.411	−0.552 to −0.270	<0.001	−0.187	−0.334 to −0.039	0.014
*Cultural support*	−0.409	−0.573 to −0.244	<0.001	−0.288	−0.446 to −0.130	<0.001

^a^ Multivariable models are adjusted for gender, age, understaffed department, shift work, and work experience. ^b^ R^2^ for the multivariable model = 3.1%; *p*-value for ANOVA = 0.017. ^c^ R^2^ for the multivariable model = 11.6%; *p*-value for ANOVA < 0.001. ^d^ R^2^ for the multivariable model = 19.9%; *p*-value for ANOVA < 0.001.

**Table 3:** Linear regression models with innovative behavior as the dependent variable (N = 328).

Dependent Variable *Independent Variables*	Univariate Model			Multivariable Model ^a^		
	Unadjusted Coefficient Beta	95% CI for Beta	*p*-Value	Adjusted Coefficient Beta	95% CI for Beta	*p*-Value
Idea generation ^b^						
*Managerial support*	0.195	0.111 to 0.280	<0.001	0.163	0.072 to 0.253	<0.001
*Organizational support*	0.134	0.044 to 0.224	0.004	0.021	−0.075 to 0.117	0.670
*Cultural support*	0.218	0.117 to 0.320	<0.001	0.181	0.078 to 0.284	0.001
Idea search ^c^						
*Managerial support*	0.127	0.032 to 0.221	0.009	0.119	0.016 to 0.222	0.023
*Organizational support*	0.070	−0.029 to 0.170	0.164	0.013	−0.097 to 0.122	0.821
*Cultural support*	0.101	−0.013 to 0.215	0.083	0.081	−0.036 to 0.198	0.175
Idea communication ^d^						
*Managerial support*	0.245	0.147 to 0.343	<0.001	0.207	0.107 to 0.306	<0.001
*Organizational support*	0.184	0.080 to 0.288	0.001	0.048	−0.057 to 0.154	0.369
*Cultural support*	0.211	0.091 to 0.331	0.001	0.149	0.036 to 0.262	0.010
Implementation starting activities ^e^						
*Managerial support*	0.267	0.162 to 0.373	<0.001	0.221	0.112 to 0.330	<0.001
*Organizational support*	0.219	0.108 to 0.331	<0.001	0.081	−0.036 to 0.197	0.173
*Cultural support*	0.222	0.093 to 0.351	0.001	0.147	0.023 to 0.272	0.020
Involving others ^f^						
*Managerial support*	0.183	0.090 to 0.276	<0.001	0.154	0.057 to 0.252	0.002
*Organizational support*	0.165	0.067 to 0.263	0.001	0.082	−0.022 to 0.186	0.122
*Cultural support*	0.093	−0.021 to 0.207	0.110	0.028	−0.083 to 0.139	0.624
Overcoming obstacles ^g^						
*Managerial support*	0.267	0.165 to 0.368	<0.001	0.216	0.107 to 0.324	<0.001
*Organizational support*	0.221	0.114 to 0.329	<0.001	0.104	−0.012 to 0.219	0.079
*Cultural support*	0.108	−0.018 to 0.234	0.094	0.028	−0.096 to 0.152	0.652

^a^ Multivariable models are adjusted for gender, age, understaffed department, shift work, and work experience. ^b^ R^2^ for the multivariable model = 9.8%; *p*-value for ANOVA < 0.001. ^c^ R^2^ for the multivariable model = 2.3%; *p*-value for ANOVA = 0.045. ^d^ R^2^ for the multivariable model = 20.2%; *p*-value for ANOVA < 0.001. ^e^ R^2^ for the multivariable model = 16.5%; *p*-value for ANOVA < 0.001. ^f^ R^2^ for the multivariable model = 12.3%; *p*-value for ANOVA < 0.001. ^g^ R^2^ for the multivariable model = 11.3%; *p*-value for ANOVA < 0.001.

**Table 4:** Linear regression models with innovation outputs as the dependent variable (N = 328).

Dependent Variable *Independent Variables*	Univariate Model			Multivariable Model ^a^		
	Unadjusted Coefficient Beta	95% CI for Beta	*p*-Value	Adjusted Coefficient Beta	95% CI for Beta	*p*-Value
Innovation outputs ^b^						
*Managerial support*	0.254	0.169 to 0.340	<0.001	0.230	0.144 to 0.317	<0.001
*Organizational support*	0.160	0.067 to 0.252	0.001	0.033	−0.059 to 0.126	0.476
*Cultural support*	0.085	−0.022 to 0.193	0.119	0.015	−0.084 to 0.113	0.771

^a^ Multivariable model is adjusted for gender, age, understaffed department, shift work, and work experience. ^b^ R^2^ for the multivariable model = 22.3%; *p*-value for ANOVA < 0.001.

## Data Availability

Our data are available from the corresponding author on reasonable request.

## Public Involvement Statement

No public involvement in any aspect of this research.

## Guidelines and Standards Statement

This manuscript was drafted against the (STROBE) for a cross-sectional study, descriptive research.
